# Screening For Pulmonary Hypertension With Multidetector Computed Tomography Among Patients With Severe Aortic Stenosis Undergoing Transcatheter Aortic Valve Implantation

**DOI:** 10.3389/fcvm.2018.00063

**Published:** 2018-06-05

**Authors:** Crochan J. O'Sullivan, Matteo Montalbetti, Rainer Zbinden, David J. Kurz, Alain M. Bernheim, Aaron Liew, Matthias R. Meyer, David Tüller, Franz R. Eberli

**Affiliations:** ^1^Department of Cardiology, Stadtspital Triemli, Zurich, Switzerland; ^2^Zurich University Hospital, Zurich, Switzerland; ^3^Department of Endocrinology, National University of Ireland, Galway, Ireland

**Keywords:** aortic stenosis, pulmonary hypertension, computed tomography, right heart catheterization, hemodynamics, transcatheter aortic valve implantation

## Abstract

**Aim:** To assess the accuracy of multi-detector computed tomography (MDCT) derived pulmonary vessel measurements in predicting pulmonary hypertension (PH) among patients with severe symptomatic aortic stenosis (AS) undergoing transcatheter aortic valve implantation (TAVI).

**Background:** PH is common among patients with severe AS undergoing TAVI and is associated with adverse outcomes. MDCT is the imaging modality of choice to assess anatomical dimensions among patients selected for TAVI.

**Methods:** One hundred and thirty-nine patients with severe AS undergoing TAVI with both CT scans and right heart catheterizations (RHC) were included. CT diameters of the main pulmonary artery (MPA), right (RPA) and left (LPA), and ascending aorta (AA) were measured. The relationship between CT measurements and PA pressures assessing using RHC was tested with linear regression.

**Results:** The CT derived ratio of the diameter of the MPA to the diameter of the AA (PA/AA_ratio_) correlated best with mean PA pressure (*R*^2^ = 0.48) and PA systolic pressure (*R*^2^ = 0.50). Receiver operating characteristic curve analysis showed that the PA/AA_ratio_ is a moderate predictor of PH (AUC 0.74, 95% CI 0.65–0.83, *p* < 0.0001) and that the optimal cut off point is 0.80 (sensitivity 56%, specificity 88%, positive predictive value 95.5%, negative predictive value 30.6% for PH).

**Conclusions:** Elderly patients with severe AS and PA/AA_ratio_ values ≥ 0.80 on MDCT are more likely to have PH but PH cannot be reliably excluded among such patients with lower PA/AA_ratio_ values.

## Introduction

Transcatheter aortic valve implantation (TAVI) is a less invasive alternative treatment option to surgical aortic valve replacement (SAVR) among patients with symptomatic severe aortic stenosis (AS) (1). Patients selected for TAVI tend to be inoperable or high risk for conventional SAVR and typically have a high prevalence of co-morbidities including coronary artery disease, atrial fibrillation, concomitant valvular heart disease, and chronic renal failure (2). In addition, pulmonary hypertension (PH) is common among patients with severe AS undergoing TAVI and is associated with worse clinical outcomes as compared with patients without PH (3). The identification of PH prior to TAVI is therefore important for appropriate risk stratification and may help in determining which patients should be selected for TAVI vs. SAVR. Right heart catheterization is the gold standard method for diagnosing PH, which is defined as a mean pulmonary artery pressure ≥25 mmHg (4). However, right heart catheterization is an invasive procedure and is not routinely performed prior to TAVI. Transthoracic echocardiography can provide an estimate of the pulmonary artery systolic pressure but cannot reliably detect whether PH is present or not (5). Multi-detector computed tomography (MDCT) is recommended as the imaging modality of choice prior to TAVI to determine annular and aortic root dimensions as well as iliofemoral anatomy prior to TAVI (6). Whether or not MDCT measurements of the pulmonary vasculature provide a reliable estimate of the presence of PH among patients with severe AS selected for TAVI is unknown. Therefore, the aim of this study was to assess the accuracy of MDCT derived pulmonary vessel measurements in predicting the presence of PH among patients with severe symptomatic AS undergoing TAVI.

## Methods

### Patient population

This is a retrospective analysis of prospectively collected data within a database that includes all patients with severe AS, who underwent TAVI at our institution between August 2011 and September 2015 (*n* = 184). All patients were deemed inoperable or at high risk for surgery by a multidisciplinary team consisting of invasive cardiologists and surgeons. Included in the present analysis were all patients with symptomatic severe AS, a full preprocedural right and left heart catheterization and a pre-procedural multidetector computed tomography (MDCT). Data collection was facilitated by using the nation-side prospective TAVI registry (SWISS TAVI Registry) into which all patients from our institution are prospectively enrolled. The cohort study complies with the Declaration of Helsinki, was approved by the local Ethics Committee, and all patients provided informed written consent.

### Cardiac catheterization

All included patients underwent coronary angiography and right and left heart catheterization for haemodynamic assessment prior to TAVI. Intracardiac pressures were recorded with fluid filled catheters connected to pressure transducers as previously described (7).

### Right heart pressures

(PH) was defined as a mean pulmonary artery pressure ≥ 25 mmHg and was subdivide into pre-capillary PH (left-ventricular end-diastolic pressure ≤ 15 mmHg) and post-capillary PH (LVEDP >15 mmHg). Furthermore, post-capillary PH was further subdivided into isolated post-capillary PH (diastolic pulmonary gradient ≤ 7 mmHg) and combined post- and pre-capillary PH (diastolic pulmonary gradient > 7 mmHg).

### Transcatheter aortic valve implantation

TAVI was performed as previously described (7). Vascular access was transfemoral using the Edwards Sapien Valve XT/S3 (ESV, Edwards Lifesciences, Irvine, CA, USA), the Medtronic CoreValve Revalveing System, the Medtronic Evolut R (MCRS; Medtronic Inc., Minneapolis, MN, USA), and the Lotus Valve (Boston Scientific) and transapical for the ESV.

### MDCT protocol and measurements for PH assessment

All included patients underwent CT for preinterventional assessment of aortic annulus size and evaluation of vascular access using a second-generation, multidetector 128-slice dual source CT (Somatom Definition Flash, Siements Healthcare, Forchheim, Germany). Images were reviewed on a stationary workstation by an investigator who had no knowledge of any clinical information or the RHC results. Calipers were set for measuring the widest short-axis diameter of the main pulmonary artery within 3 cm of the bifurcation, the right pulmonary artery and left pulmonary artery and the ascending aorta, respectively on axial sections. The diameter of the AA was measured at the level of the MPA.

### Statistics

Continuous data are presented as means ± standard deviations (SD), and categorical variables are depicted as percentages and numbers. Categorical variables were compared by means of the χ^2^ test (or Fisher's test for two group comparisons), and continuous variables were compared using the unpaired *t*-test for two groups or ANOVA for 3 or more groups. ROC analysis were performed to assess the AUC and to compare sensitivity and specificity for different cut-off values using the Youden Index. Sensitivity, specificity, negative predictive value (NPV), and positive predictive value (PPV) were calculated and shown in percentages. Time-to-event data are presented using Kaplan-Meier curves, with incidence rates calculated from life-tables at 2-year follow-up. Log-rank test was used to declare significance. A *p*-value < 0.05 were considered statistically significant. All analyses were performed with SPSS 22, Release 22.0.0.1 or STATA (version 12, StataCorp, College Station, TX, USA).

## Results

### Patient characteristics

**Figure 1:**
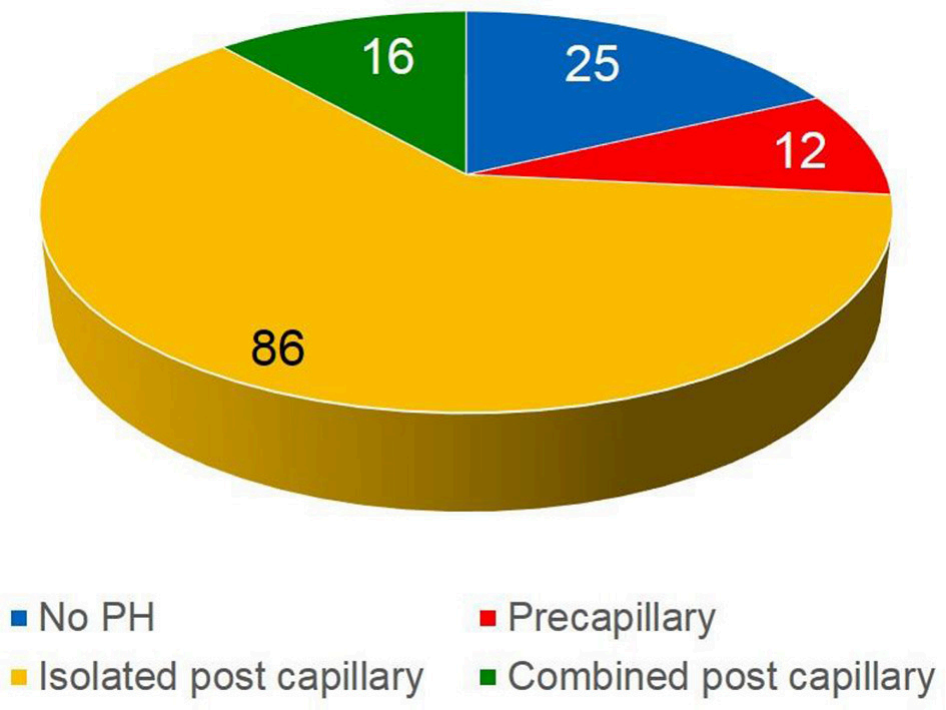
Classification of the patient population (*n* = 139).

Baseline characteristics are given in Table 1. A total of 139 patients with symptomatic severe AS undergoing TAVI had complete MDCT and RHC data and were included in the present analysis. Eighty-Two percentage (*n* = 114) patients had PH defined as a mean PA pressure ≥ 25 mmHg. PH patients comprised 12 patients with precapillary PH, 86 patients with isolated post-capillary PH and 16 patients with combined precapillary and post-capillary PH (Figure 1). Patients with PH were significantly older, had a higher body mass index and had significantly higher surgical risk scores at baseline. In addition, NT-pro-BNP values at baseline were significantly higher among PH patients as compared with no PH patients (Table 1) (*p* < 0.0001).

**Table 1:** Baseline characteristics.

	**All patients *N* = 139**	**No PH *N* = 25**	**PH *N* = 114**	***P*-value**
**DEMOGRAPHICS**				
Age (years)	83.58 ± 4.98	81.70 ± 3.97	84.0 ± 5.10	0.04
Female gender, n (%)	59 (42.4)	13 (52.0)	46 (40.4)	0.20
**PHYSICAL DIMENSIONS**				
Height (cm)	165.58 ± 7.81	167.06 ± 8.16	165.06 ± 7.67	0.09
Weight (kg)	71.24 ± 15.76	67.40 ± 15.01	72.09 ± 15.85	0.18
Body mass index (kg/m2)	25.93 ± 5.33	23.81 ± 4.72	26.39 ± 5.37	0.03
BSA (m2)	1.81 ± 0.23	1.77 ± 0.23	1.82 ± 0.23	0.30
**CARDIAC RISK FACTORS**				
Diabetes mellitus, n (%)	27 (19.4)	4 (16.0)	23 (20.2)	0.44
Hypercholesterolemia, n (%)	30 (21.6)	8 (32.0)	22 (19.3)	0.13
Hypertension, n (%)	92 (66.2)	15 (60.0)	77 (67.5)	0.31
**PAST MEDICAL HISTORY**				
Coronary artery disease, n (%)	64 (46.0)	12 (48)	52 (45.6)	0.50
Previous myocardial infarction, n (%)	11 (7.9)	3 (12.0)	8 (7.0)	0.31
Previous coronary artery bypass graft, n (%)	12 (8.6)	1 (4.0)	11 (9.6)	0.32
Previous percutaneous coronary intervention, n (%)	23 (16.5)	4 (16.0)	19 (16.7)	0.60
Previous cerebrovascular event, n (%)	19 (13.7)	6 (24)	13 (11.4)	0.10
Peripheral vascular disease, n (%)	32 (23.0)	5 (20.0)	27 (23.7)	0.46
Chronic obstructive pulmonary disease, n (%)	15 (10.8)	3 (12.0)	12 (10.5)	0.53
Previous pacemaker, n (%)	16 (11.5)	2 (8.0)	14 (12.3)	0.42
Renal failure (GFR < 60 ml/min/1.73 m^2^)	99 (71.2)	17 (68.0)	82 (71.9)	0.43
**HEART RHYTHM**				
Atrial fibrillation, n (%)	34 (24.5)	4 (16.0)	30 (26.3)	0.26
**SYMPTOMS**				
Syncope, n (%)	15 (10.9)	4 (16.0)	11 (9.7)	0.28
**New york heart association (NYHA) functional class**				
*NYHA III/IV*, n (%)	114 (82.0)	18 (72.0)	96 (84.2)	0.13
**Canadian cardiovascular society (CCS) angina status**				
*CCS III/IV*, n (%)	9 (6.5)	2 (8.0)	7 (6.1)	0.51
**RISK ASSESSMENT**				
Logistic EuroScore (%)	20.71 ± 13.25	14.20 ± 10.73	22.16 ± 13.36	0.01
STS Score (%)	4.95 ± 2.84	3.94 ± 2.13	5.17 ± 2.93	0.05
**LABORATORY VALUES**				
HS Troponin T (ng/ml)	30.72 ± 25.00	22.17 ± 21.52	32.59 ± 25.41	0.06
NT-Pro Brain Natriurtic Peptide (pg/mL)	3,338 ± 3,831	1,554 ± 1,774	3,749 ± 4,058	< 0.0001

### Echocardiographic and invasive haemodynamic characteristics

Baseline echocardiographic and invasive haemodynamic characteristics are shown in Tables 2, 3, respectively. As compared with no PH, patients with PH had significantly larger left ventricular end-diastolic diameters (*p* = 0.026), higher non-invasive right ventricular/right atrial gradients (*p* = 0.026), higher left ventricular end-diastolic pressures (*p* = 0.005), higher pulmonary and right ventricular pressures (*p* < 0.0001) and lower pulmonary artery saturation measurements (*p* = 0.026).

**Table 2:** Baseline echocardiography characteristics.

	**All patients *N* = 139**	**No PH *N* = 25**	**PH *N* = 114**	***P*-value**
**AORTIC STENOSIS SEVERITY**				
Aortic valve area (cm^2^)	0.71 ± 0.20	0.75 ± 0.22	0.70 ± 0.19	0.43
Aortic maximal velocity, cm/s	4.3 ± 0.69	4.0 ± 0.4	4.3 ± 0.7	0.16
Mean gradient (mmHg)	46.6 ± 14.0	47.3 ± 12.5	46.5 ± 14.4	0.83
Peak gradient (mmHg)	72.0 ± 21.1	70.1 ± 17.4	72.2 ± 21.9	0.75
**LV GEOMETRY AND 2D MEASUREMENTS**				
LV end-systolic diameter, mm	32.5 ± 11.1	26.3 ± 12.3	34.0 ± 10.5	0.06
LV end-diastolic diameter, mm	45.3 ± 10.4	40.0 ± 12.4	46.6 ± 9.4	0.026
LV mass index, g/m^2^	131.69 ± 33.0	121.7 ± 52.1	133.7 ± 27.7	0.49
**LV SYSTOLIC FUNCTION**				
LV ejection fraction, %	56.1 ± 12.9	58.8 ± 10.0	55.4 ± 13.5	0.35
**ASSOCIATED VALVULAR ABNORMALITY**				
Aortic regurgitation				0.74
None	58 (41.7)	11 (44.4)	47 (41.2)	
Mild	58 (41.7)	8 (32.0)	50 (43.9)	
Moderate	5 (3.6)	1 (4.0)	4 (3.5)	
Severe	3 (2.2)	1 (4.0)	2 (1.8)	
Mitral regurgitation				0.30
None	33 (23.7)	7 (28.0)	26 (22.8)	
Mild	62 (44.6)	14 (56.0)	48 (42.1)	
Moderate	32 (23.0)	2 (8.0)	30 (26.3)	
Severe	3 (2.2)	0 (0)	3 (2.6)	
Tricuspid regurgitation				
None	48 (34.5)	9 (36.0)	39 (34.2)	0.86
Mild	63 (45.3)	10 (40.0)	53 (46.5)	
Moderate	15 (10.8)	3 (12.0)	12 (10.5)	
Severe	2 (1.4)	0 (0)	2 (1.8)	
**RIGHT SIDED HEMODYNAMICS**				
RV-RA gradient, mmHg	38.2 ± 13.5	28.0 ± 6.4	40.4 ± 13.7	0.026

**Table 3:** Invasive haemodynamic characteristics.

	**All patients *N* = 139**	**No PH *N* = 25**	**PH *N* = 114**	***P*-value**
**AORTIC STENOSIS SEVERITY**				
Aortic valve area (cm^2^)	0.64 ± 0.30	0.72 ± 0.36	0.63 ± 0.29	0.19
Peak-to-peak gradient (mmHg)	54.95 ± 26.03	50.48 ± 23.96	55.88 ± 36.45	0.37
Mean gradient (mmHg)	44.74 ± 18.24	41.42 ± 17.25	45.44 ± 18.44	0.34
**SYSTEMIC VASCULAR LOAD**				
Systolic arterial pressure (mmHg)	138.34 ± 28.65	132.32 ± 26.04	139.66 ± 29.14	0.25
Diastolic arterial pressure (mmHg)	66.10 ± 14.39	66.04 ± 11.71	66.11 ± 14.96	0.98
Mean arterial pressure	95.09 ± 17.31	92.88 ± 14.11	95.58 ± 17.95	0.48
Systemic vascular resistance (mmHg.min.L^−1^)	*1, 839*±*761*	*1, 729*±*578*	*1, 861*±*792*	0.47
**LV SYSTOLIC FUNCTION**				
Ejection fraction (%)	53.80 ± 14.30	56.87 ± 13.21	53.15 ± 14.50	0.26
LV systolic pressure (mmHg)	197.10 ± 38.37	186.87 ± 37.30	199.22 ± 38.41	0.16
LV end diastolic pressure (mmHg)	26.13 ± 9.57	21.13 ± 7.23	27.17 ± 9.69	0.005
Stroke volume (ml)	57.36 ± 23.61	63.43 ± 21.25	56.17 ± 23.96	0.20
Stroke volume index (ml/m2)	32.01 ± 12.16	35.51 ± 10.82	31.32 ± 12.33	0.15
Cardiac output (L/min)	4.12 ± 1.35	4.47 ± 1.25	4.05 ± 1.36	0.19
Cardiac index (l/(min*m2)	2.30 ± 0.68	2.50 ± 0.59	2.26 ± 0.69	0.14
Heart rate (beats/min)	75.82 ± 16.97	71.08 ± 14.71	76.86 ± 17.31	0.12
**RIGHT SIDED HEMODYNAMICS**				
PA systolic pressure (mmHg)	53.38 ± 16.72	33.72 ± 5.09	57.69 ± 15.22	< 0.0001
PA diastolic pressure (mmHg)	22.37 ± 8.53	12.72 ± 3.42	24.49 ± 7.83	< 0.0001
Mean PA pressure (mmHg)	35.76 ± 11.57	21.28 ± 2.59	38.94 ± 10.27	< 0.0001
RV systolic pressure, mmHg	53.82 ± 15.19	36.64 ± 6.67	57.62 ± 13.86	< 0.0001
RA mean pressure (mmHg)	9.38 ± 4.89	5.68 ± 2.72	10.17 ± 4.90	< 0.0001
Diastolic pressure gradient ≥ 7 mmHg, n (%)	26 (18.8)	1 (0.7)	25 (21.9)	0.031
**COMPONENTS OF FICK EQUATION**				
Aortic saturation (%)	92.72 ± 3.88	94.01 ± 3.18	92.45 ± 3.97	0.07
Pulmonary artery saturation (%)	60.62 ± 11.08	65.18 ± 6.81	59.66 ± 11.58	0.026
Hemoglobin (g/dl)	12.43 ± 1.49	12.75 ± 1.34	12.36 ± 1.53	0.51

### Procedural characteristics

Procedural characteristics are shown in Table 4. The majority of patients underwent TAVI via the transfemoral route (78%) under general anesthesia (91%) with the Edwards SAPIEN 3 valve (35%). Balloon predilatation was performed in most cases (86%) and only a minority of patients underwent concomitant revascularization.

**Table 4:** Procedural characteristics.

	**All patients**	**No PH**	**PH**	***P*-value**
	***N* = 139**	***N* = 25**	***N* = 114**	
Access route				0.42
Femoral, n (%)	109 (78.4)	22 (88.0)	87 (76.3)	
Apical, n (%)	29 (20.9)	3 (12.0)	26 (22.8)	
Direct aortic, n (%)	1 (0.7)	0 (0)	1 (0.9)	
Valve type				0.83
Edwards Sapien 3, n (%)	49 (35.3)	8 (32.0)	41 (36.0)	
Edwards Sapien valve XT, n (%)	31 (22.3)	5 (20.0)	26 (22.8)	
Medtronic CoreValve, n (%)	42 (30.2)	9 (36.0)	33 (28.9)	
Medtronic Evolut R, n (%)	10 (7.2)	1 (4.0)	9 (7.9)	
Boston Scientific Lotus, n (%)	7 (5.0)	2 (8.0)	5 (4.4)	
Anesthesia				0.15
General, n (%)	127 (91.4)	21 (84.0)	106 (93.0)	
Local, n (%)	12 (8.6)	4 (16.0)	8 (7.0)	
Balloon predilation				0.76
Balloon predilation	119 (85.6)	21 (84.0)	98 (86.0)	
Revascularisation				0.37
Revascularisation, n (%)	5 (3.6)	0 (0)	5 (4.4)	
Procedural Specifications				0.74
Post-preocedure moderate-severe AR, n (%)	17 (12.2)	2 (8.0)	15 (13.2)	

### MDCT measurements referring to PH

MDCT measurements of the pulmonary and aortic vasculature are shown in Tables 5, 6. As compared with no PH, patients with PH had significantly larger diameters of MPA (*p* = 0.001), RPA (*p* = 0.004), LPA (*p* = 0.029), and PA/AA_ratio_ (*p* < 0.0001). No significant differences in ascending aorta or aortic annular measurements were observed between groups. As compared with no PH, patients with combined post-capillary PH had significantly larger MPA (*p* = 0.006), RPA (*p* = 0.011) diameters and PA/AA_ratio_ (*p* < 0.0001) (Supplementary Table 1). In addition, patients with precapillary PH had significantly larger LPA and RPA diameters as compared with patients without PH (supplementary Table 1).

**Table 5:** Computer Tomography characteristics.

	**All patients *N* = 139**	**No PH *N* = 25**	**PH *N* = 114**	***P*-Value**
**PULMONARY VASCULAR DIAMETERS**				
Main pulmonary artery, mm	28.01 ± 4.33	26.03 ± 2.92	28.44 ± 4.48	0.001
Right pulmonary artery, mm	27.25 ± 4.08	25.13 ± 4.47	27.71 ± 3.86	0.004
Left pulmonary artery, mm	25.70 ± 3.07	24.49 ± 3.24	25.96 ± 2.98	0.029
Main pulmonary artery/ascending aorta ratio	0.80 ± 0.12	0.73 ± 0.08	0.82 ± 0.12	< 0.0001
**AORTIC ANNULUS MEASUREMENTS**				
Ascending aorta, mm	35.11 ± 4.21	36.06 ± 4.17	34.91 ± 4.21	0.21
Annulus perimeter, mm	89.22 ± 14.81	91.03 ± 14.87	88.82 ± 14.90	0.63
Annulus area, mm^3^	483 ± 115	529 ± 138	472 ± 107	0.11

**Table 6:** Diagnostic accuracy of computed tomography for detecting pulmonary hypertension.

	**Sensitivity (%)**	**Specificity (%)**	**NPV (%)**	**PPV (%)**
MPA ≥ 29 mm	39.5	84.0	23.3	91.8
MPA ≥ 30 mm	28.9	84.0	20.6	89.2
MPA ≥ 31 mm	24.6	100.0	22.5	100.0
MPA ≥ 32 mm	19.3	100.0	21.4	100.0
MPA ≥ 29 mm (only men)	47.8	76.9	29.4	88.0
MPA ≥ 31 mm (only men)	30.4	100.0	28.9	100.0
MPA ≥ 27 mm (only females)	52.9	83.3	23.8	94.7
MPA ≥ 29 mm (only females)	33.8	91.7	19.6	95.8
MPA ≥ 31 mm (only females)	20.6	100.0	18.2	100.0
PA/AA ratio ≥ 0.75	69.3	56.0	28.6	87.8
PA/AA ratio ≥ 0.80	56.1	88.0	30.6	95.5
PA/AA ratio ≥ 0.85	36.8	96.0	25.0	97.7
PA/AA ratio ≥ 0.90	28.1	100.0	23.4	100.0
PA/AA ratio ≥ 0.95	14.9	100.0	20.5	100.0
PA/AA ratio ≥ 0.1.0	4.4	100.0	18.7	100.0

*AA, ascending aorta; MPA, main pulmonary artery*.

**Figure 2:**
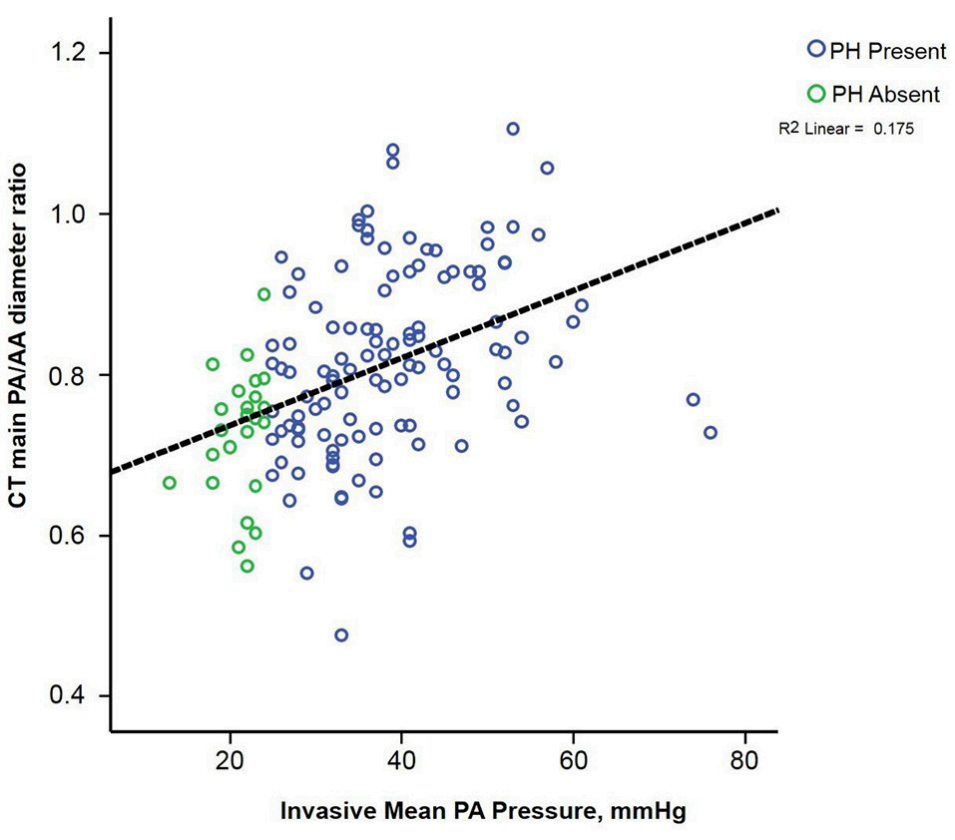
Scatterplot illustrating the correlation between the CT derived pulmonary artery/ascending aorta ratio.

**Figure 3:**
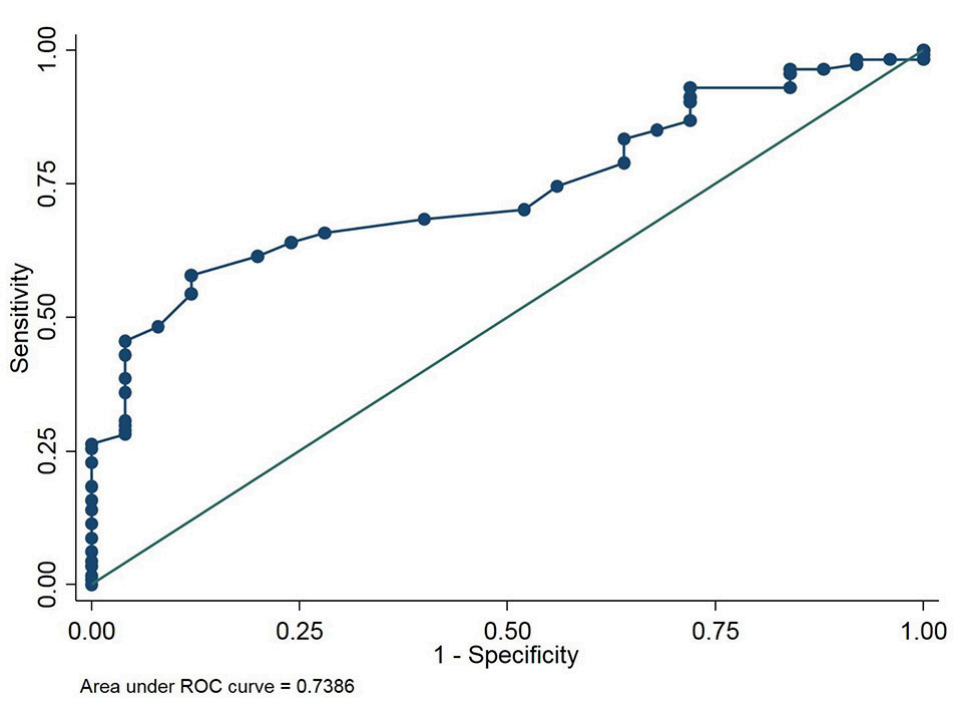
Receiver operating characteristic curve testing the ability of the CT derived pulmonary artery/ascending aorta ratio to detect PH defined as a mean pulmonary artery pressure ≥ 25 mmHg.

**Figure 4:**
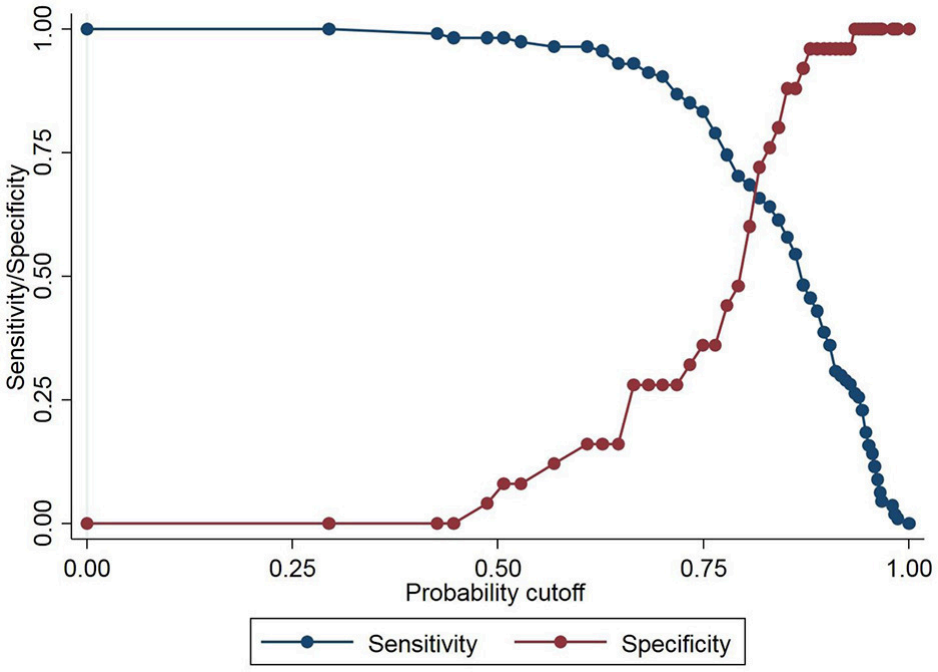
Graph illustrating the best cut-off point for the CT derived pulmonary artery/ascending aorta ratio to detect PH. The best cutoff derived from the Youden index was 0.80.

The PA/AA_ratio_ exhibited the best correlation with PA pressures (Figure 2) (*r*^2^ 0.48 for mean PA, *p* < 0.0001; *r*^2^ 0.50 for PA systolic pressure, *p* < 0.0001; *r*^2^ 0.41 for diastolic PA pressure, *p* < 0.0001). Using Receiver Operating Characteristic Curves, the PA/AA_ratio_ correlated best with PH (AUC 0.74), whereas MPA (AUC 0.65), RPA (AUC 0.67), and LPA (AUC 0.64) exhibited lower sensitivity and specificity (Figure 3). The optimal cut-off point of the PA/AA_ratio_ in predicting the presence of PH defined as an invasive mean PA pressure is 0.80 with a sensitivity of 56%, specificity of 88%, negative predictive value of 30.6%, and positive predictive value of 95.5% (Figure 4).

### Clinical outcomes at 30-Days and 2-Years

**Figure 5:**
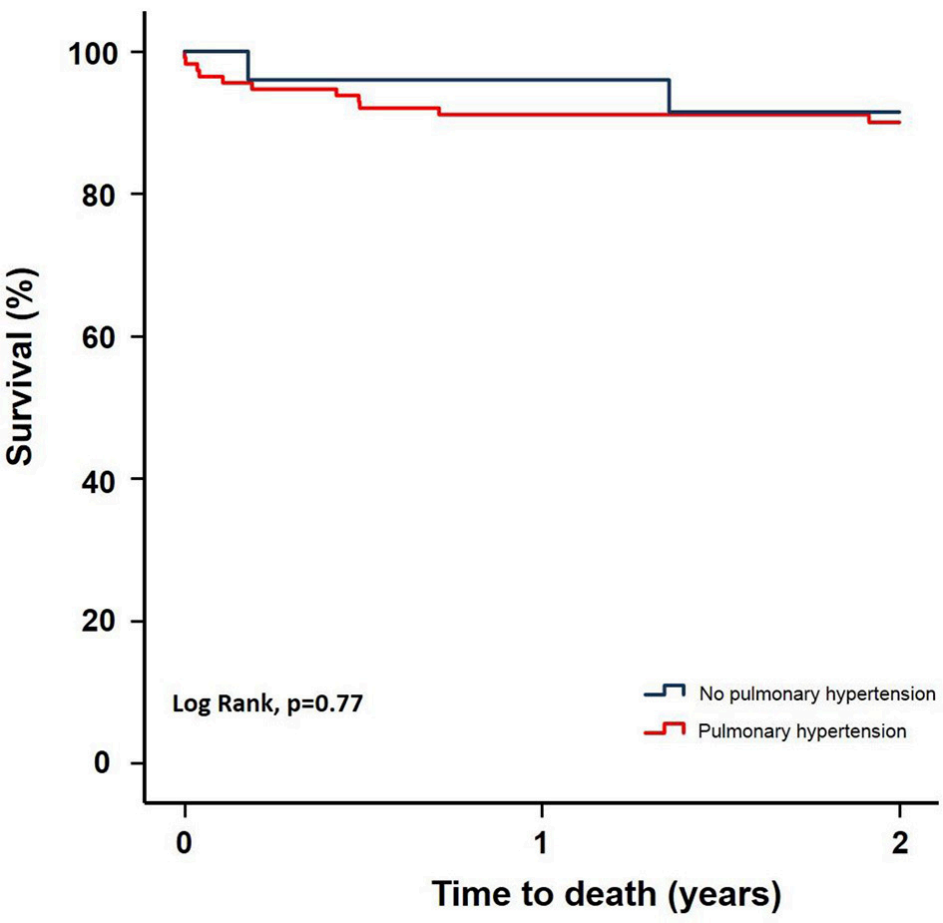
Kaplan-Meier survival curve for patients with and without PH.

Clinical outcomes at 30-days and 2-years are shown in Table 7. As compared with no PH, no significant differences in all-cause mortality (Hazard Ratio 0.80, 95% confidence interval 0.18–3.60, *p* = 0.77) or cardiovascular mortality (HR 4.29, 95% CI 0.60–30.45, *p* = 0.11) were observed at 2 years among patients with PH (Figure 5). In addition, no significant differences in other VARC-2 endpoints (cerebrovascular accidents, major bleeding, vascular complications, acute renal failure, and permanent pacemaker implantation) were observed between groups at 30-days or 2 years (Table 7).

**Table 7:** Clinical outcomes.

	**No PH *N* = 25**	**PH *N* = 114**	***P*-value**
**30 DAYS FOLLOW-UP**			
All cause death, n (%)	0 (0)	4 (3.5)	0.45
Cardiovascular death, n (%)	0 (0)	0 (0)	
Cerebrovascular events	1 (4.0)	0 (0)	0.18
Major stroke, n (%)	0 (0)	0 (0)	
Minor stroke, n (%)	0 (0)	0 (0)	
Transient ischemic attack, n (%)	1 (4.0)	0 (0)	0.18
Bleeding	4 (16.0)	19 (16.7)	0.60
Life-threatening, n (%)	1 (4.0)	3 (2.6)	0.55
Major, n (%)	1 (4.0)	7 (6.1)	0.56
Acute renal failure, n (%)	0 (0)	1 (0.9)	0.82
Access site complications	3 (12.0)	9 (7.9)	0.37
Major, n (%)	1 (4.0)	4 (3.5)	0.64
Minor, n (%)	2 (8.0)	5 (4.4)	0.37
New permanent pacemaker, n (%)	4 (16.0)	15 (13.2)	0.46
**2 YEAR FOLLOW-UP**			
All cause death, n (%)	2 (8.0)	11 (9.6)	0.58
Cardiovascular death, n (%)	2 (8.0)	2 (1.8)	0.15
Cerebrovascular events	1 (4.0)	4 (3.5)	0.64
Major stroke, n (%)	0 (0)	2 (1.8)	0.67
Minor stroke, n (%)	0 (0)	1 (0.9)	0.82
Transient ischemic attack, n (%)	1 (4.0)	1 (0.9)	0.33
All cause death or major stroke, n (%)	2 (8.0)	13 (11.4)	0.47

## Discussion

In the present study we sought to assess the reliability of screening for PH using MDCT derived measurements of the pulmonary arteries as compared with gold standard pulmonary artery pressure measurements derived from right heart catheterization among patients with severe AS undergoing TAVI. The key finding was that PA/AA_ratio_ was the most useful parameter to use to screen for PH among patients with severe AS undergoing TAVI with moderate to high specificity but relatively low sensitivity. We found that the best PA/AA_ratio_ cutoff for screening for PH is 0.80. Therefore, patients with a larger PA/AA_ratio_ on CT are more likely to have PH (high positive predictive value) but PH cannot be reliably ruled out among patients with smaller PA/AA_ratio_ values (low negative predictive value). PH is common among patients with severe AS selected for TAVI and is associated with worse clinical outcomes as compared with patients without PH (3, 8–10). Right heart catheterization is the gold standard method for diagnosing PH but is not routinely performed prior to TAVI. Conversely, MDCT is almost always performed prior to TAVI in order to assess aortic annular and vascular dimensions for procedural planning (6). Consequently, MDCT may serve as a useful screening tool for the presence of PH among patients selected to undergo TAVI and help with risk stratification.

### CT derived anatomical indicators of PH

Truong et al. determined the age and sex specific distribution and normal reference values for main pulmonary artery diameter and the PA/AA_ratio_ by CT in an asymptomatic community-based population (*n* = 3,171, mean age 51 ± 10 years) (11). The investigators observed the 90th percentile cutoff value for PA/AA_ratio_ was 0.90 for both males and females but that the PA/AA_ratio_ was inversely proportional to age (11). The authors observed that that the PA/AA_ratio_ was smaller in older participants owing to progressive aortic enlargement with increasing age (11). In the present study, the mean age of the study sample was 84 ± 5 years and this may account for the fact that we observed a smaller PA/AA_ratio_ of 0.80 to be the optimal predictor of PH. Prior studies in younger patients with more heterogeneous diseases have suggested that a PA/AA_ratio_ ≥ 1.0 is the optimal cutoff point to diagnose PH on CT. A small retrospective study (*n* = 50, median age 47.5 years) found that a PA/AA_ratio_ > 1.0 was the best predictor of chronic pulmonary arterial hypertension with a sensitivity, specificity and positive and negative predictive values for PH of 70, 92, 96, and 52% (12). Similary, Sanal et al. observed in a retrospective study among patients with pulmonary embolism (mean age 59 ± 15 years) that a PA/AA_ratio_ ≥ 1.0 had a 59% sensitivity, 82% specificity, a 55% positive predictive value, and a 84% negative predictive value for diagnosing moderate to severe PH defined as a pulmonary artery systolic pressure ≥ 50 mmHg on Doppler Echo (13). Mohamed Hoesein et al. (14) assessed the accuracy of CT PA diameter and PA/AA_ratio_ for PH in end-stage COPD among 92 patients (mean age 55 years) and found that a PA/AAratio >1 had a negative predictive value of 77.9% and a positive predictive value of 63.1%. However, the results of the present study would suggest that in an elderly patient population, such as those selected to undergo TAVI, a PA/AA_ratio_ ≥ 1.0 would not be sensitive enough as we observed a sensitivity of just 4.4% when PA/AA_ratio_ ≥ 1.0 was used to predict the presence of PH. Therefore, the key observation of this study is that a lower PA/AA_ratio_ value is required to screen for PH among TAVI patients. To date only one other study assessed the value of CT pulmonary vascular measurements as a predictor of PH and mortality in symptomatic severe AS (15). In contrast to the present study, the authors did not observe that PA/AA_ratio_ was any better at predicting PH as compared with MPA. The reasons for this are unclear but may relate to the fact that the pulmonary artery measurements may have been made during end-systole rather than end-diastole. The investigators also did not find any significant differences between MPA diameters of patients with combined post-and pre-capillary PH and no PH, whereas we observed significant differences between these groups (15).

### Limitations

The present study is a single center retrospective study with several limitations. Although we observed no significant differences in adverse clinical outcomes between patients with and without PH no definitive conclusions on the effect of PH on mortality can be drawn from this study as too few events occured. However, the main aim of this study was not to compare clinical outcomes between patients with and without PH, but rather to assess the accuracy of CT measurements of the pulmonary vessels in predicting the presence of PH. The presented conclusions are preliminary and only hypothesis generating and that further research is needed. Further studies should test whether we are able to diagnose different severities of PH, since the implications of severe PH are completely different from mild.

## Conclusions

In the present study we found that PA/AA_ratio_ demonstrates the strongest correlation with mean PA and PA systolic pressures and that the optimal cutoff is 0.80 in predicting the presence of PH with high specificity but moderate to low sensitivity.

## Author contributions

CO, MM, RZ, DK, and FE: conception and design of the study. CO and MM: analysis and interpretation of data. CO: drafting of the manuscript. All authors revising the manuscript critically for important intellectual content and final approval of the manuscript submitted.

### Conflict of interest statement

The authors declare that the research was conducted in the absence of any commercial or financial relationships that could be construed as a potential conflict of interest.
