# Supplementary material for: Screening For Pulmonary Hypertension With Multidetector Computed Tomography Among Patients With Severe Aortic Stenosis Undergoing Transcatheter Aortic Valve Implantation
Source: Front Cardiovasc Med. 2018 Jun 5;5:63. doi: 10.3389/fcvm.2018.00063 (PMC6008561; doi:10.3389/fcvm.2018.00063)
Supplement: Supplementary file 1 [file Table_1.PDF]

Supplementary Table 1. MDCT characteristics according to pulmonary hypertension subgroup

	No PH<br>N=25	Precapillary PH<br>N=12	Isolated PH<br>N=86	Combined PH<br>N=16	p-value
MPA, mm	26.04±2.92	29.35±5.88	27.86±4.01	30.93±5.04*	0.006
RPA, mm	25.13±4.48	29.09±4.25*	27.37±3.71	28.51±4.24*	0.011
LPA, mm	24.49±3.24	27.72±3.18*	25.81±2.80	25.48±3.45	0.024
PA/AA Ratio	0.73±0.08	0.80±0.15	0.81±0.12*	0.88±0.12*	<0.0001

LPA left pulmonary artery; MPA main pulmonary artery; PA/AA Ratio pulmonary artery/ascending aorta ratio; RPA right pulmonary artery. * Significant difference as compared with no PH.
